# Phytohormonal modulation of the drought stress in soybean: outlook, research progress, and cross-talk

**DOI:** 10.3389/fpls.2023.1237295

**Published:** 2023-10-20

**Authors:** Shifa Shaffique, Saddam Hussain, Sang-Mo Kang, Muhamad Imran, Md. Injamum-Ul-Hoque, Muhammad Aaqil Khan, In-Jung Lee

**Affiliations:** ^1^ Department of Applied Biosciences, Kyungpook National University, Daegu, Republic of Korea; ^2^ Department of Agronomy, University of Agriculture, Faisalabad, Pakistan; ^3^ Biosafety Division, National Institute of Agriculture Science, Rural Development Administration, Jeonju, Republic of Korea; ^4^ Department of Chemical and Life Science, Qurtaba University of Science and Information Technology, Peshawar, Pakistan

**Keywords:** phytohormones, drought stress, soybean, microbes, endogenous

## Abstract

Phytohormones play vital roles in stress modulation and enhancing the growth of plants. They interact with one another to produce programmed signaling responses by regulating gene expression. Environmental stress, including drought stress, hampers food and energy security. Drought is abiotic stress that negatively affects the productivity of the crops. Abscisic acid (ABA) acts as a prime controller during an acute transient response that leads to stomatal closure. Under long-term stress conditions, ABA interacts with other hormones, such as jasmonic acid (JA), gibberellins (GAs), salicylic acid (SA), and brassinosteroids (BRs), to promote stomatal closure by regulating genetic expression. Regarding antagonistic approaches, cytokinins (CK) and auxins (IAA) regulate stomatal opening. Exogenous application of phytohormone enhances drought stress tolerance in soybean. Thus, phytohormone-producing microbes have received considerable attention from researchers owing to their ability to enhance drought-stress tolerance and regulate biological processes in plants. The present study was conducted to summarize the role of phytohormones (exogenous and endogenous) and their corresponding microbes in drought stress tolerance in model plant soybean. A total of n=137 relevant studies were collected and reviewed using different research databases.

## Graphical Abstract

A schematic representation of the phytohormone modulates drought stress tolerance where **(A)** represents the endogamous phytohormone modulation, **(B)** represents the activation of phytohormones via application of the microorganism **(C)** represents the exogenous application of phytohormones regulates the drought stress tolerance.

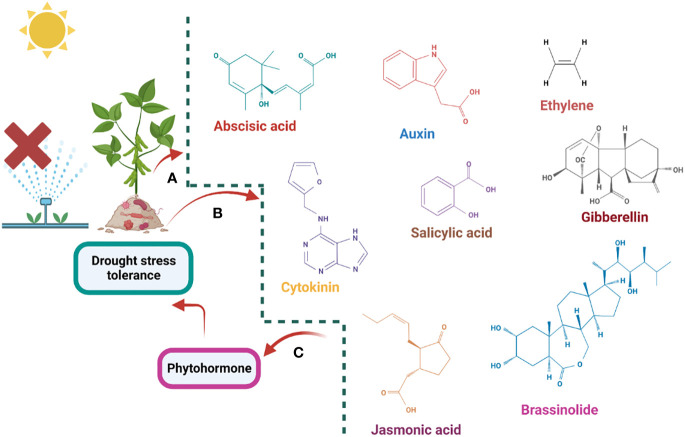

## 1 Introduction

**Figure 1:**
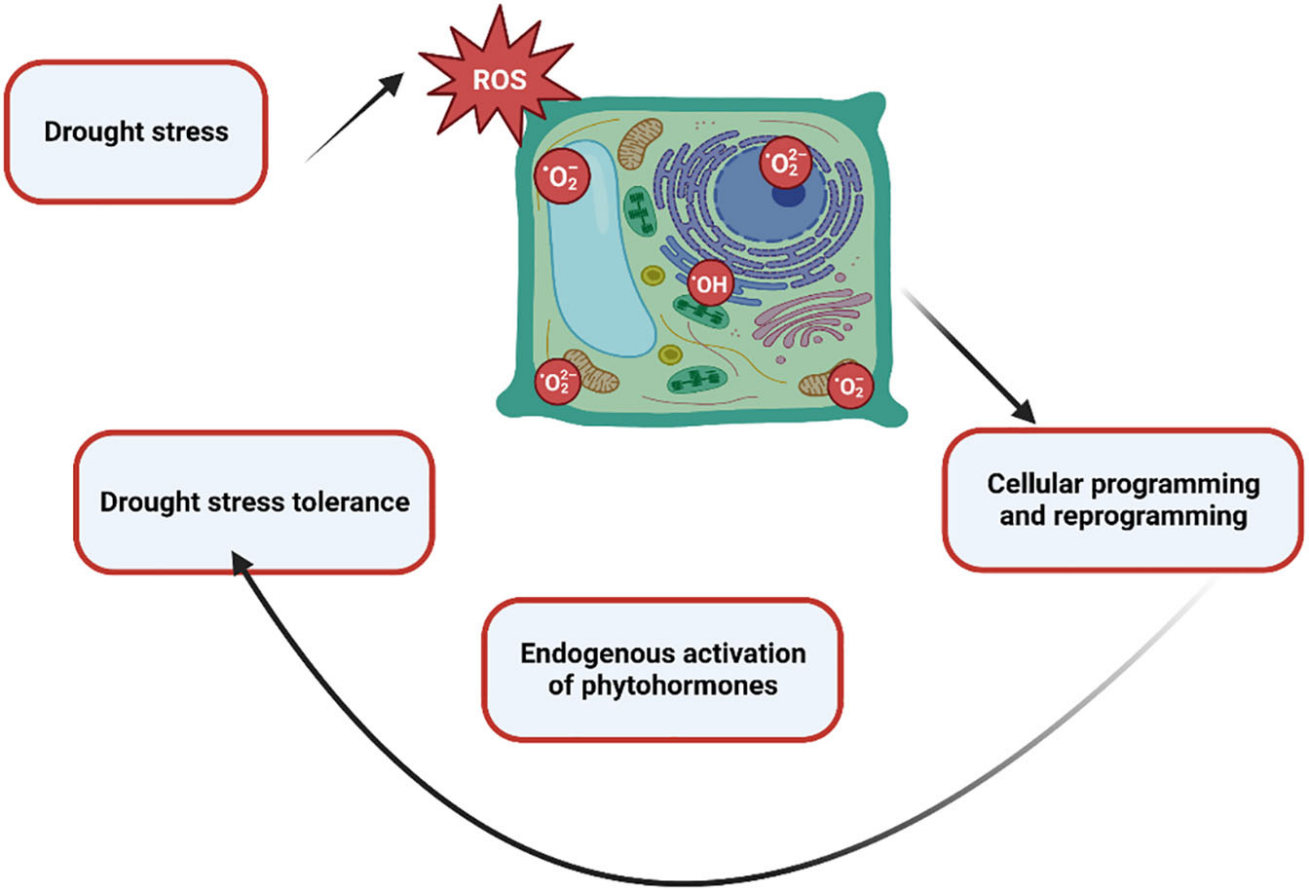
A holistic view of the mechanism of oxidation stress and drought stress tolerance represents the plant’s cellular reprogramming via activation of the phytohormones to modulate the drought stress.

**Figure 2:**
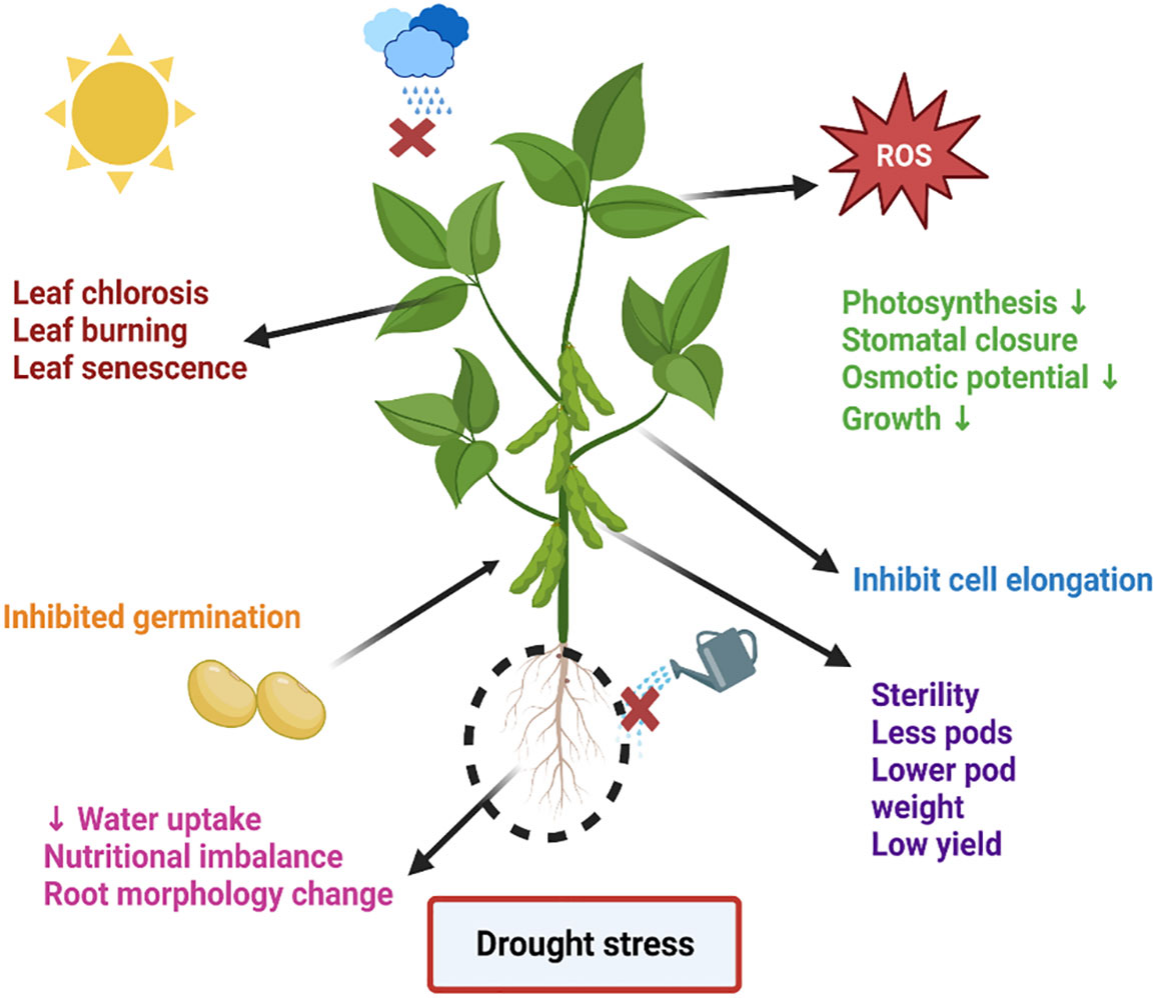
An overview of drought stress and its symptoms. The figure represents the symptoms appearing on the soybean after drought stress.

Environmental stress negatively affects plant productivity by up to 70%. Various ecological stresses, including biotic and abiotic stresses, hinder plant development (Mittler, 2006; Choudhury et al., 2017). The agricultural industry is under double pressure. One is environmental stress, and the other is population growth. It is predicted that the global population will reach 10 billion by 2050. Furthermore, by 2050, agricultural crops will have lost up to 30% of their production. In addition, the heat index will reach 52 degrees Celsius. Among abiotic stresses, drought stress poses an alarming risk to agronomical yield, minimizing plant yield and productivity. Moreover, it is a multidimensional stress that arrests plant biomass and energy at molecular and sub-molecular levels. Changes in the climate and landscape temperature, increasing population, and shortage of rain in a particular period enhance the intensity of drought stress (Hasanuzzaman et al., 2013; Shaffique et al., 2022a). One of the main abiotic stresses that has a negative impact on crop growth and production is water deficiency. These modifications are mostly linked to changed metabolic processes, such as reduced or absent photosynthetic pigment production, ion uptake and translocation, glucose biosynthesis, food metabolism, and growth promoter synthesis. The generation of reactive oxygen species (ROS) in response to plant stress is directly correlated with these modifications to metabolic processes and the creation of photosynthetic pigments. Reductions in fresh and dry biomass are a frequent detrimental outcome of water stress on crop plants (Batool et al., 2022; Madouh and Quoreshi, 2023). Strong relationships exist between the mechanisms of dry matter partitioning and temporal biomass distribution and plant productivity under drought stress. Numerous biochemical mechanisms, such as the fluidity of plasma membranes, the production of osmolytes, lipid peroxidation, the generation of (ROS), the rigidity of cellular membranes, and the activation of various enzymes involved in the oxidative defense system, are all triggered by drought stress. In the past, the production of ROS in different crop species has caused serious harm to proteins, lipid peroxidation (LPO), and other cellular components (Ajithkumar and Panneerselvam, 2014). The lipid membrane and protein were catastrophically affected by drought stress-induced ROS production. The majority of ROS are created during photosynthesis by enzymatic or non-enzymatic mechanisms, including the superoxide radical (O^-2^), hydrogen peroxide (H_2_O_2_), singlet oxygen (^1^O_2_), and hydroxyl radical (OH^-^). Additionally, they are produced by partial oxidation or reduction in the mitochondrial electron transport system components (Impa et al., 2012). The control of ROS homeostasis involves a number of cellular events that plants use to withstand oxidative stress. As byproducts of several metabolic processes in diverse cellular compartments like chloroplast, mitochondria, and peroxisomes, plants continuously produce a variety of free radicals. Their efficient scavenging by enzymatic and non-enzymatic cascades typically counter balances the formation of ROS in plant cells. ROS have partially reduced forms of ambient oxygen. Because ROS can harm various biomolecules, including DNA, proteins, and lipids, it can result in oxidative injury, which inhibits plant growth and development. In response to the free radicals oxidation stress increases. However, plant cells reprogram the cellular event via activation of the phytohormones and antioxidant (**Figure 1**) to mitigate the stress up to a certain limit after that cell shows symptoms of drought stress such as the burning of the leaf, necrosis, fewer pods, lower weight pods, reduced productivity, lower yield crops, inhibit germination, reduced water potential and closure of the stomata to prevent the loss of water etc., (Xiong et al., 2020). As shown in **Figure 2**.

In 1970, scientists discovered certain chemicals that can optimize plant growth from seed germination to plant development, which were subsequently termed chemical messengers or phytohormones. The concentration of phytohormones required to optimize plant growth is low (10^–6^ to 10^–5^ mol/L), thereby making it difficult to study these phytohormones (Tarakhovskaya et al., 2007; Su et al., 2017). After 1970, research involving phytohormones and their interactions and implications in plant physiology progressed. All phytohormones exhibit distinctive features, nature, and location (Lu and Xu, 2015; Pozo et al., 2015). Their roles have been extensively elucidated not only in plant growth and development but also in stress mitigation (Khan et al., 2012; Egamberdieva et al., 2017). Phytohormones are natural organic s molecules that coordinate with one another to regulate complex signaling pathways and ensure optimal functioning of cellular plant activities (Javid et al., 2011; Fenn and Giovannoni, 2021). Phytohormones exhibit diverse characteristics; for example, ethylene is an alkene, abscisic acid (ABA) is a sesquiterpenoid, gibberellins (GAs) are tetracyclic diterpenoid, jasmonic acid (JA) is a derivative of linoleic acid, and brassinosteroids (BRs) are steroids (Kefeli et al., 2003; Altmann et al., 2020). Endogenous plant hormones are important in the response to drought, in addition to supporting signaling pathways. Plants’ responses to osmotic adjustment under stress are heavily mediated by phytohormones. Small signaling molecules known as phytohormones have a substantial impact on nearly every aspect of plant development (Sati et al., 2023; Shaffique et al., 2023b). Furthermore, it is generally understood that a single hormone can influence a wide range of cellular and developmental processes or that numerous hormones can regulate a single function at the same time. Plants are protected and controlled by phytohormones against biotic and abiotic stressors. As a result, the use of phytohormones seeks to broaden agricultural stress research in the future (Singh and Roychoudhury, 2023).

Exogenous phytohormone treatment is a more promising technique for dealing with the negative impacts of drought on sustainable agriculture production. Because of their multi-functionality against abiotic stressors, phytohormones are gaining popularity among plant researchers (Ozturk and Unal, 2023; Swain et al., 2023). However, their use in legume crops (Soybean) is still limited. As a result, the current work expands the use of phytohormones on model plant soybean under drought stress. Deep insights into the physiological, biochemical, and molecular basis of soybean adaptation to drought were also investigated.

Several studies have shown that microbes produce small amounts of phytohormones that enhance plant growth and stress tolerance in several plant species. Numerous studies have documented the usefulness of phytohormone-producing microorganisms in reducing abiotic stress in plants (Shaffique et al., 2022a; Shaffique et al., 2023a). **Table 1** lists some examples of phytohormone-producing bacteria and their capacity to lessen abiotic stress. Numerous studies have documented the beneficial benefits of plant-associated bacteria and IAA generation on promoting plant growth under abiotic stress situations. For example, the bacterial isolate *Enterobacter ludwigii* SH-6 increased plant biomass and improved drought stress tolerance in maize (Shaffique et al., 2022b).

**Table 1:** Phytohormone-producing bacteria and their action mechanism in drought-stress tolerance in soybean.

Country, Year, and Reference	Strain	Phytohormones	Mechanism of action (MOA)
South Korea 2014 (Kang et al., 2014)	*Pseudomonas putida H-2-3*	ABA GA	Modulating antioxidant defense system
India 2019 (Vaishnav and Choudhary, 2019)	*Pseudomonas simiae AU*	ABA	Genetic expression Overexpression of *DREB*, *PIP*, and *TIP*
South Korea 2019 (Bilal et al., 2020)	*LHL10 and LHL06*	ABA GA	Over expression of gene *DREB*, Modulating antioxidant defense system SOD CAT Glutathione,
Brazil 2019 (Bulegon et al., 2019)	*Azospirillum brasilense*	CK	↑RWC ↑Grain yield ↑Gas exchange
2010 South Korea (Khan et al., 2011)	*LH02*	ABA GA SA	Secondary metabolites↑
Iran 2015 (Zahedi and Abbasi, 2015)	*Rhizobium japonicum, Azotobacter chroococcum* and *Azospirillum brasilense*	GA ABA	Growth promotion

The ↑ and ↓ represents the increase and decrees of the specific response.

Soybean constitutes an important legume crop that is a rich source of nutrition. Owing to its nutritional value, soybean is in high demand in developing countries. It is rich in protein, oils, and fibers (Specht et al., 2014; Novikova et al., 2022; Razgonova et al., 2022). The soybean (*Glycine max*) is a crop that is produced all over the globe and is considered an essential ingredient in cuisine due to the large amount of nutrients it contains. Even though there have been considerable improvements in production, environmental stress remains a persistent danger for the soybean crop. There has not been enough research on the soybean, particularly about the modulatory role of phytohormones modulation in the model plant soybean. There is a significant gap in studies between phytohormones and soybean. Environmental stresses, particularly drought stress, considerably affect soybean production (Li et al., 2019; Feng et al., 2020). Herein, we explored the research progress made regarding the effect of phytohormones in mitigating drought stress in soybean. The importance of phytohormones as plant growth regulators have led to a new era of research focusing on phytohormones producing biostimulants to enhance drought-stress tolerance. We further highlighted that phytohormone-producing microbes can mitigate drought stress. We searched the terms “phytohormone, drought, and soybean” on the search engine and downloaded and studied relevant articles for a comprehensive review. There were certain inclusion and exclusion criteria applied to the articles included in this review. All relevant data, including the nature and action mechanism of the phytohormones and gas chromatography–mass spectrometry (GCMS), liquid-chromatography–mass spectrometry (LCMS), chromatographic quantification of hormone analysis and hormonal role, and exogenous application data, published until 2022 were included. Non-English data and conference papers were excluded.

## 2 Review

Phytohormones govern all cellular functions in higher plants and serve an important role in organizing various signal transduction pathways during plant stress response. Their critical function in promoting adaptability to ever-changing environments through growth and differentiation, changes in the source/sink ratio, and nutrient allocation has been well described (Paul et al., 2023; Wani et al., 2023). Abiotic stressors activate signal transduction cascades that are linked to baseline pathways transduced by plant hormones. Aside from the five conventional phytohormones auxins, cytokinins, gibberellins (GA), abscisic acid (ABA), and ethylene (ET), newly found phytohormones include salicylic acid (SA), brassinosteroids, jasmonate (JA), polyamines, and strigolactone. Abscisic acid, salicylic acid, jasmonate, and ethylene are well-known for their anti-inflammatory properties. According to (Zheng et al., 2023), abscisic acid, salicylic acid, jasmonate, and ethylene all contribute positively to plants’ ability to withstand stress. For the control of plant defensive responses, auxins, cytokinin, and gibberellins interact with ABA, ethylene, salicylic acid, and jasmonate. The organization of various genes and their regulators involved in stress relief is made possible by hormonal cross-talk. Therefore, it is crucial to comprehend the intricate relationship between cross-talk among phytohormones. Cross-talk between jasmonate and salicylic acid exists, which can interact either negatively or positively by assisting in the development of specific defense responses. At various points along the signaling pathways that result in the control of salicylic acid and jasmonate (Ahmad et al., 2023; Singh and Roychoudhury, 2023)

### 2.1 Phytohormone abscisic acid triggers drought stress tolerance

ABA is a sesquiterpenoid with the molecular formula C_15_H_2_O_4_. It has various biological functions and is present in organisms belonging to numerous kingdoms, such as mosses, algae, plants, cyanobacteria, and mammals (Seo and Koshiba, 2002; Kuromori et al., 2018). ABA constitutes an important growth regulator of plant cells. In addition, an important phytohormone controls plant productivity and stress tolerance (Muhammad Aslam et al., 2022). ABA is sometimes referred to as the stress hormone. Reactive oxygen species quickly rise when a plant is under stress, amplifying oxidative stress (Murtaza et al., 2016).

Endogenous ABA levels rise immediately after oxidative stress. ABA acts as a signaling molecule. The ABA involves the regulation of stress-responsive genes, for example, dehydration-responsive element-binding (*DREB*) proteins and basic leucine zipper (*bZIP*), during drought stress to improve plant productivity via a stress tolerance mechanism (Liang et al., 2011). A genome-wide association study (GWAS) of soybean plants under stress revealed that ABA either induces or suppresses more than half the genes. The ABA-induced genes included regulatory proteins, transcription factor (Tf), kinases, and phospholipase enzymes for signaling pathways. The ABA-suppressed genes promoted plant growth by regulating guard cells, stomatal conductance, root development, and photosynthesis (Hirayama and Shinozaki, 2007; Yoshida et al., 2014). Stomatal conductance via guard cell regulation is vital for drought stress mitigation in plants. When plants are exposed to drought stress, guard cells regulate osmosis by activating genes that encode proteins to prevent dehydration in all the plant cells (Raghavendra et al., 2010; Ma et al., 2018). Furthermore, ABA intervenes in the high root length density under osmotic stress conditions to reach water present at deeper levels. In addition, ABA modulates root architecture in various ways, including lateral root formation and adaptive morphological alterations, such as reduced xylem diameter to promote the axial hydraulic conductivity of soil under water scarcity stress (Danquah et al., 2014; Sakata et al., 2014).

Six independent studies included herein involved the application of ABA in soybean, and their findings confirmed that exogenous ABA application improved drought stress tolerance in the plant. Initially, independent studies were conducted in 2004 to investigate the effects of exogenous ABA application on soybean during the early reproductive stage. The results indicated that stress exposure increased the ABA levels up to 1.5-fold and decreased the plant’s photosynthetic activity. Exogenous ABA application decreased the pod set owing to its direct effect on the metabolic processes in the ovary; however, further studies regarding the mechanism underlying this effect are warranted (Liu et al., 2004). The second independent study indicated that exogenous ABA application rapidly overexpressed the *G_m_RAV* gene, which is involved in several plant biological processes, including the signaling pathway, to form a complex network in drought stress tolerance (Zhao S.-P. et al., 2017). Exogenous ABA application also improves plants’ relative water content (RWC) and root-to-shoot ratio. For this study, two drought genotypes were selected: tolerant and susceptible genotypes. A comparison of the genotypes revealed that the drought-tolerant genotype was more likely to respond to ABA than the drought-susceptible genotype (Xing et al., 2016). Suinong 14 (*Glycine max*) was experimentally analyzed under different drought conditions to evaluate the effectiveness of ABA on drought-stress tolerance. Drought stress was induced naturally by withholding water from 69.0 MPa to 35.0 MPa at the flowering stage. Subsequently, 1.0–8.0 mg/L ABA was applied exogenously. All secondary metabolites were screened and excluded. The results indicated that up to 2.0 mg/L ABA enhanced soybean’s antioxidant capability and drought tolerance (Ruan et al., 2012). This result was further confirmed by another study with similar findings. Furthermore, exogenous ABA application improved osmotic adjustment and leaf water potential but not plant yield (He et al., 2019).

Inoculating plants with plant growth–promoting rhizobacteria (PGPR) mediates ABA production and drought-stress mitigation by activating several relevant cascades. The proposed action mechanism involves ABA-mediated osmotic adjustment regulation and turgor pressure maintenance (Kour and Yadav, 2022).A scientific report by Kang et al. (2014) published in Korea revealed that *Pseudomonas putida* H-2-3 mitigated drought stress by endogenously producing ABA. Supposedly, ABA is a stress-responsive hormone that is upregulated during stress, and it serves as a signaling molecule to activate the antioxidant defense system in soybean (Kang et al., 2014). In 2019, Vaishnav et al. reported that the *Pseudomonas simiae* strain AU mitigated drought stress by regulating the endogenous hormone, ultimately activating various gene expressions, such as those of *DREB* and water transporter genes, including *PIP* and *TIP*, which are involved in osmotic stress regulation (Vaishnav and Choudhary, 2019). The results are given in **Table 1**.

### 2.2 Jasmonic acid

JA, a chief plant growth regulator, modulates biological processes in plants. It is a derivative of α-linoleic acid, and jasmonate is an active derivative of JA. In response to abiotic stresses, such as drought, heat, chilling and heavy metals, JA enhances stress tolerance in plants. Furthermore, it promotes plant growth (Muñoz-Espinoza et al., 2015; Ghorbel et al., 2021; Kim et al., 2021). The primary features of JA that distinguish it from other hormones include its involvement in fruit ripening, pollen production, tendril coiling, and root hydraulic pressure (Ruan et al., 2019; Wang et al., 2020). The complete genomic analysis of soybean demonstrated that various JA-regulated genes confer stress tolerance. The action mechanism of JA is similar to that of ABA, as both hormones induce stomatal closure. Furthermore, various studies have described that exogenous JA application induces stomatal closure in soybean (Stintzi et al., 2001; Du et al., 2013; Ghorbel et al., 2021; Wang et al., 2021).

Stress-induced endogenous phytohormone production is a multifarious spectacle that involves several enzymatic pathways (Kaur and Asthir, 2017). Free radical accumulation under drought stress conditions converts unsaturated fatty acid into 12-oxo-phytodienoic acid (12-OPDA) and deoxymethylated vegetable dienic acid in peroxisomes. Furthermore, free radicals convert endogenous JA into their corresponding molecules in peroxisomes. Following the conversion, JA and its derivatives, including MeJA, JA-Ile,12-hydroxyjasmonic acid, and cis-jasmone, enter the cytoplasm to further confer free radicals (Koo et al., 2009; Savchenko et al., 2014; Kim et al., 2017).12-OPDA is a JA precursor, and various studies have confirmed that its concentration is inversely proportional to the stomatal aperture and stress tolerance of plants. Stress prevents the conversion of 12-OPDA to jasmonate. This delayed conversion might be related to ABA, which induces stress tolerance via stomatal closure (Hu et al., 2009; Ali and Baek, 2020). The findings of several studies have evidenced that exogenous JA application induces stomatal closure, further enhancing the production of antioxidant molecules such as dehydroascorbate acid reductase (DHAR), ascorbate, glutathione reductase (GR), and monodehydroascorbate reductase (MDAR), and consequently, plant stress tolerance. In addition, JA accumulation in roots increases ABA concentration (de Ollas et al., 2015). Therefore, ABA and JA function synergistically to enhance plant stress tolerance (Dathe et al., 1981; Seo et al., 2001; de Ollas et al., 2013).

A pot experiment was performed to elucidate the effectiveness of methyl jasmonate on the stress tolerance of soybean. The exogenous application of 50 µM methyl jasmonate inhibited leaf gas exchange and plant growth and reduced chlorophyll content under extreme drought stress (Anjum S. A.et al., 2011). Jasmonate is involved in a natural molecular signaling pathway that regulates plant progress under stress conditions. Furthermore, it enhances ABA production to control plants’ stomatal conductance and water status (Ghassemi-Golezani and Farhangi-Abriz, 2021). Conversely, some studies have reported that exogenous JA application enhances secondary metabolite production, inhibits trypsin, and induces protective genes against plant environmental stresses (Anjum S. et al., 2011). Another research report suggested that 50 µM methyl jasmonate progresses drought-stress tolerance by minimizing lipid peroxidation (LPO) and enhancing the free radicals scavenging system (Hao L. et al., 2013).

In 2014, Kyungpook National University, a leading university in Korea considered the hub of investigating plant microbial interactions, informed that inoculating soybean with *P. putida* H-2-3 enhances the levels of JA and downregulates those of ABA and SA. The results indicated that *P. putida* H-2-3 reprograms chlorophyll and improves hormonal regulation, thereby mitigating drought stress in plants (Kang et al., 2014) In 2019, entophytic fungi LHL10 and LHL06 were reported to synergistically improve drought-stress tolerance by downregulating JA levels synergistically, overexpressing the Gm*DREB* gene, and modulating the Intrinsic defense system by production of antioxidant enzymes as shown in **Table 1** (Bilal et al., 2020).

### 2.3 Cytokinins

Like other hormones, cytokinins (CKs) are biologically involved in the process of plant growth, development, and stress acclimatization (Werner et al., 2001; Kieber and Schaller, 2014). The chief distinguishing feature of CKs is that it promotes stress acclimatization and adaptation alongside tolerance. Cytokinins play numerous roles in plant growth and morphogenesis. They regulate cell division and interact with auxins to control apical dominance, lateral branching, and the root-shoot ratio in intact plants and tissue culture. They slow leaf senescence and promote dark-grown seedlings’ light-independent deetiolation response, including greening. Plants naturally produce a number of cytokinins. They have an adenine base and an isopentenyl side chain of five carbons. Zeatin, specifically trans-zeatin, is the most abundant of them. The concreted functions of isopentenyl transferase, CK oxidase, and CK dehydrogenase maintain the CK hemostatic level in plant cells. Plants are multicellular organisms with complex networks of interactions among its hormones. Recently, it was established that plants exhibit a sophisticated coping mechanism in response to stress involving the CK signal transduction pathway (Ha et al., 2012; O’Brien and Benková, 2013). CKs play a dual role in the stress signaling pathway (Pospíšilová et al., 2000; Kakimoto, 2003); they serves as a negative regulator by inducing the expressions of certain genes such as CKX1, CKX2, CKX3, and CKX4, thereby acclimatizing the plant during stress (Brugière et al., 2003; Niemann et al., 2018), and a positive regulatory effect by increasing the CK levels and delaying senescence in plants (Mok, 2019). When CKs (zeatin riboside) were exogenously applied, they restored the germination potential in *Glycine max* seeds (Gidrol et al., 1994; Gupta et al., 2000).The inoculation of *Azospirillum brasilense* in soybean mitigates drought stress by inducing the production of endogenous phytohormones, for example CKs. The results indicated that CKs improve the RWC by 76.96%, gas exchange by up to 860.43%, and grain yield by 19% (Bulegon et al., 2019).

### 2.4 Gibberellins

GAs are tetracyclic diterpenoid carboxylic acids essential for plant biological functions and stress tolerance. The exact mechanism underlying GA-mediated drought-stress tolerance modulation remains unknown (Takahashi et al., 2012; Hedden and Sponsel, 2015). However, reportedly, GAs exhibit a positive effect on plants throughout their life cycle, promoting cell elongation and division during the juvenile and adult stages (Tanimoto, 2002; Yamaguchi, 2008). GA may also negatively regulate plant stress tolerance by inhibiting its biosynthesis. Furthermore, previous studies have reported that GA content was reduced under water scarcity stress (Fleta-Soriano et al., 2015; Omena-Garcia et al., 2019). Many studies have found that gibberellins significantly improve plant drought tolerance (Iqbal et al., 2022). Drought-responsive element-binding protein (*DREB*) improves drought tolerance by lowering the expression of gibberellin biosynthesis genes. Reduced GA levels in plants are claimed to improve drought tolerance (Wang et al., 2017; Yang et al., 2021). An environmentally friendly plant growth regulator used all around the world is mepiquat chloride (DPC). DPC is frequently employed to regulate plant geometry in addition to maintaining a balance between nutrition and reproductive growth. The biosynthesis and signal transduction of other plant hormones, such as zeatin and brassinolide, as well as the production and signal transduction of GA were altered by DPC. Additionally, DPC encouraged the formation of flavonoids, increasing drought resistance (Wang et al., 2022).

Similar to other hormones, the exogenous application of GA also recovers the physio-metabolic features of plants and enhances their stress tolerance. Numerous studies have reported that the application of GA hormones enhances plant productivity and mitigates drought stress (Cohen et al., 2009).

In 2011, (Khan et al., 2011) described that the Inoculation of LH02 in soybean improved its stress tolerance by inducing GA and secondary metabolite production. In 2014, (Kang et al., 2014) experimentally observed the effect of *P. putida* H-2-3 strain on the drought-stress tolerance of soybean. The results revealed that this microbial strain enhanced the GA levels by modulating the antioxidant status and enhancing the plant’s stress tolerance by up to 15%.In 2015, three-plant growth–promoting rhizobacteria, namely *Rhizobium japonicum*, *Azotobacter chroococcum*, and *A. brasilense*, were inoculated into the soybean plant. Drought stress was induced naturally via water scarcity. The hormonal status and physio-morphological features of the plant were studied. The results showed that inoculation enhanced drought-stress tolerance by modulating the levels of GAs and ABA (Zahedi and Abbasi, 2015). In 2019, (Bilal et al., 2020) reported that the entophytic fungi LH10 and LH06 synergistically improved drought-stress tolerance by producing up to 300 ng/g GAs. Moreover, the application of these strains downregulated the DRE-binding Tf and decreased ABA levels and oxidation stress in plants.

### 2.5 Salicylic acid

SA is an essential endogenous phytohormone that regulates protein expression and contributes to the plant defense system. Similar to the other hormones, including ABA and JA, SA is also involved in plant stress tolerance. SA overexpression in response to drought stress is due to two inducible genes, namely PR1 and PR2 (Hayat and Ahmad, 2007; Chen et al., 2009). Salicylic acid (SA) is a key regulator of immunity and programmed cell death in plants. According to early research, greater SA accumulation during resistance gene-mediated defense responses is linked to the initiation of the hypersensitive reaction. In lesion-mimic mutants, SA was also discovered to accumulate to high levels, and in certain cases, this accumulation is necessary for the phenotype of spontaneous cell death. High amounts of SA have been demonstrated to inhibit plant cell death during effector-triggered immunity, indicating that SA has two roles in the regulation of cell death. The drought stress-induced activation of such genes produces a protective effect in plants. The levels of SA may increase up to many folds in response to stress (Shah, 2003; Rao et al., 2012); particularly, exposure to drought stress increases the SA levels and promotes stomatal closure by generating free radicals(Ilyas et al., 2017; Noreen et al., 2017). However, the effect of SA on drought-stress tolerance in plants remains debatable. Some researchers have reported that SA positively regulates drought-stress tolerance, whereas others have claimed the opposite, thereby rendering its actual effect controversial (Janda et al., 2007; Khan et al., 2015). A small concentration (0.5 mM) of SA induces drought tolerance, whereas a higher concentration (2–3 mM) enhances stress (Ben Ahmed et al., 2009; Zhao P. et al., 2017). SA application causes ROS generation in the chloroplast, thereby reducing stress tolerance via a cascade of events, including antioxidant activation and hormonal modulation. Thus, SA is an important hormone involved in plant stress tolerance. The findings of various studies have evidenced that SA application enhances stress tolerance (Khan et al., 2018).

When inoculated into *G. max*, the *Pseudomonas simiae* strain AU mitigates drought stress by producing phytohormones, particularly SA, and upregulating the Tf of *DREB*, osmoprotactans, and water transporter genes (Vaishnav and Choudhary, 2019).

### 2.6 Brassinosteroids

BRs are mainly plant steroids based phytohormones involved in regulating plant physiological development. They regulate photosynthesis, photo-morphogenesis, seed germination, fertility, flowering, fruit ripening, grain filling, and leaf senescence (Krishna, 2003; Zhu et al., 2013). They also play an important role in stress tolerance. 24-epibrasonolide EBR is a BR derivative. The action mechanism of BR involves increasing the efficiency of light consumption by the photosystem II (Divi and Krishna, 2009; Hao J. et al., 2013).

Exogenous BR application improves plant photosynthetic characteristics, RWC, and antioxidant enzyme production by reducing hydrogen peroxide and monoaldehyde contents (Shahbaz and Ashraf, 2007; Soares et al., 2020). However, to the best of our knowledge, no studies have implicated microbial BR production in enhancing drought-stress tolerance in soybean. Only a few studies have suggested that BRs are emerging growth regulators that can be used in sustainable agricultural practices. However, although they exhibit a promising effect on plant growth, the role of BRs in stress tolerance remains debatable (Nolan et al., 2017; Alhaithloul and Soliman, 2021).

### 2.7 Auxins

Auxin, indole acetic acid (IAA), is an important plant hormone that regulates several biological processes, from seed dormancy to development. It plays a primitive role in stress mitigation. Drought stress in plants induces a rapid overexpression of *YUCCA* (YUC) (Woodward and Bartel, 2005; Zhao, 2010), which belongs to the flavin monooxygenase protein family responsible for auxin biosynthesis (Overvoorde et al., 2010; Sauer et al., 2013). The overexpression of this gene family is attributed to apical dormancy, which tall the stem like slender and increases plant drought tolerance. Auxins promote drought tolerance by modulating root architecture (Zarea, 2019; Fadiji et al., 2022). Furthermore, they function in synergy with ABA. Thus, the overexpression of ABA-responsive genes causes ROS accumulation and auxin activation (Van Ha et al., 2013; Wu and Zhang, 2019; Li et al., 2022). Auxins are considered eco-friendly biofertilizer and can be applied in sustainable agricultural practices. Reportedly, auxins exhibit an ameliorative effect on plants that are under stress conditions (Van Ha et al., 2013).

In 2019, (Bulegon et al., 2019) reported that *A. brasilense* inoculation improved drought-stress tolerance in plants by inducing the production of phytohormones, such as auxins, which improved crop yield by up to 19%. In 2021, three bacterial strains, namely AKAD A1-1, AKAD A1-2, and AKAD A1-16, belonging to the *Bacillus* family, were reported to produce auxins. Thus, inoculating these strains can mitigate drought stress in a soybean variety via the modulation of phytohormones (Dubey et al., 2021).

### 2.8 Ethylene

Ethylene (C_2_H_4_) is an important gaseous hormone implicated in germination, flowering, fruit ripening, senescence, and stress tolerance. It also plays a dual role in regulating stomatal conductance (Arraes et al., 2015). Osmotic stress induces ROS accumulation in plants, thereby activating ABA-induced stomatal closure. When stress is over, ethylene inhibits the ABA-induced stomatal closure (Sharp, 2002; Yan et al., 2016). Furthermore, ethylene promotes stomatal closure via NADPH oxidase accumulation (Husain et al., 2020). Under stress conditions, ROS accumulation mediates NADPH oxidase production, which promotes stomatal closure (Acharya and Assmann, 2009; Wahab et al., 2022).

Beneficial microbes mitigate drought stress by modulating certain plant hormones, such as ethylene, which is responsible for the plant stress mitigation mechanism (Rehrig, 2010; Ma et al., 2019). The downregulation of ethylene improves abiotic stress tolerance in various plants (Kim et al., 2012; Chandra et al., 2018).

## 3 Conclusion and future prospective

Plants have evolved to develop sophisticated mechanisms involving phytohormones and phytohormone-producing microbes to combat drought stress. The endogenous modulation, exogenous application of phytohormones and phytohormone-producing microbes strengthens the defense mechanism of plants, as shown in the graphical abstract. Moreover, plants synthesize diverse signaling molecules in response to drought stress. Furthermore, this review highlights that phytohormone-producing microbes enhance drought-stress tolerance in soybean, thereby providing a platform for introducing microbes that can mitigate drought stress by inducing the production of phytohormones and activating their molecular action mechanism. Microbial mediation has led to considerable progress in plant drought-stress tolerance. Although the relevant literature is encouraging in sustainable agronomy, future studies are warranted to elucidate the fundamental mechanism underlying microbe-mediated enhancement of drought-stress tolerance in plants. This review will help scientists develop sustainable agriculture production via phytohormones and their corresponding microbes. Strigolactones are sparse in the scientific literature for soybean growth under drought stress, thus scientists should pay more attention to and conduct more research on strigolactone-generating microorganisms.

## Author contributions

SS conceptualized and wrote the original draft and drew the figures. SH did the critical review editing. MI, S-MK and MK did the formatting. Prof I-JL supervised and validated the results. All authors contributed to the article and approved the submitted version.
